# Applying microbial ecology frameworks to microbial therapies for wildlife

**DOI:** 10.1128/msystems.00598-25

**Published:** 2026-04-24

**Authors:** Sally L. Bornbusch, Carly R. Muletz-Wolz

**Affiliations:** 1 Center for Conservation Genomics, Smithsonian's National Zoo and Conservation Biology Institute 189732 , Washington, DC, USA; 2 Department of Nutrition Science, Smithsonian's National Zoo and Conservation Biology Institute, Washington, DC, USA; 3 Complex Microbial Systems Group, National Institute of Standards and Technology 10833, Gaithersburg, Maryland, USA; University of Connecticut, Storrs, Connecticut, USA

**Keywords:** microbiome, wildlife, microbial ecology, conservation, microbial therapy, ecology, probiotics, fecal microbiota transplant

## Abstract

Microbial ecology is increasingly incorporated into human and animal medicine via the study and purposeful manipulation of host-associated microbiomes. Microbial therapies—treatments with the aim of beneficially modulating microbiomes—are a burgeoning area of research and industry. These microbial therapies include prebiotic dietary items, live probiotics, and whole microbiota transplants (e.g., fecal microbiota transplants). Although microbial therapies for humans and domestic animals are now widely produced for commercial use and application, evidence supporting the efficacy of commercial microbial therapies is mixed. We suggest that microbial therapies are most effective when paired with concepts from ecology and rigorous empirical research. This is particularly relevant for the development and use of microbial therapies in wildlife animal species, in which we see large-scale variation in microbial communities across hosts of varying ecologies. Identifying and developing microbial therapies that can simultaneously be accessible and effective in a variety of hosts poses a novel challenge for microbial ecologists, animal scientists, and human and animal medical professionals. In addition to pre- and probiotics, we suggest that whole microbiota transplants provide a method of microbial supplementation that may better align with species-specific microbial ecology. Moving forward, emerging methods used in human medicine such as machine learning, network analysis, and microbiome engineering using high-throughput culturomics will likely be key to identifying and applying functionally relevant (e.g., disease suppressive) microbial taxa for wildlife therapies.

## INTRODUCTION

Microbial ecology—the study of how microorganisms interact with each other and their host and/or environment—is increasingly used to advance our understanding of human and animal microbiomes. Treatments that purposefully manipulate host-associated microbiomes—hereafter referred to as microbial therapies—have revolutionized human and animal medicine. From the advent of antibiotic drugs to more recent applications of prebiotics, probiotics, and whole community transplants, such as fecal microbiota transplants (FMTs), the use of microbial therapies has rapidly expanded into human and animal health. The integration of ecology into practical microbial therapies can provide critical context for understanding and modulating host-associated microbiomes. For instance, Tian et al. recently used ecological network analysis to construct a lab-built microbial consortium and identify the functionally relevant taxa that underlie the success of FMTs in treating *Clostridioides difficile* infection in humans (1). Such ecological approaches and proposed frameworks are now prevalent in the human literature (2–5) and emerging as an area of interest in wildlife health and conservation (6–13).

Applying ecological principles and methods is relevant for the use of microbial therapies in wildlife conservation. In the face of the ongoing biodiversity crisis, preserving threatened animal species under human care offers a demographic, genetic, and potentially microbial safety net against extirpation and extinction. Yet, our ability to maintain the health of *ex situ* animals is hampered, in part, by minimal empirical research on effective microbial therapies in wildlife species. Under human care, variation in wildlife microbiome structure has been linked to gastrointestinal disease and infection (14–16), reproductive dysfunction (17–19), and pathogen-mediated morbidity and mortality during conservation efforts like translocations or reintroduction (15, 20). We emphasize that, to tackle these conservation challenges, the study and use of microbial therapies in wildlife care and conservation should be grounded in ecological concepts. In this minireview, we have three main objectives: (i) introduce ecological concepts that can provide a framework for microbial therapies, (ii) discuss how the application of that framework can advance microbial therapies for wildlife, namely prebiotics, probiotics, and fecal microbiota transplants, and (iii) present future avenues for the practical application of microbial ecology and microbial therapies to advance biodiversity conservation. We focus mainly on vertebrate species, given the bulk of literature devoted to vertebrate microbiomes and the preponderance of vertebrate wildlife under human care. Although we prioritize literature and examples in wildlife species, throughout, we pull from the significantly more robust literature on humans and domestic animals. The goal of this unique review is to provide an ecological framework that improves our ability to evaluate and use multiple microbial therapies in wildlife conservation.

## ECOLOGICAL CONCEPTS THAT CAN INFORM MICROBIAL ECOLOGY FRAMEWORKS IN WILDLIFE CONSERVATION

**Fig 1:**
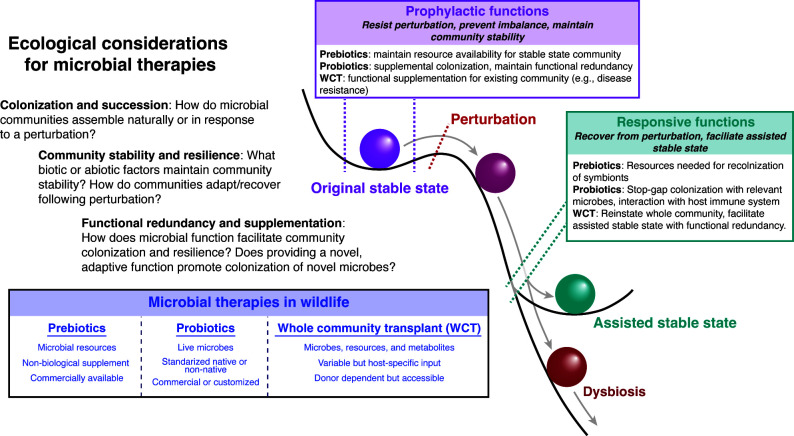
Synthesized framework for incorporating ecological principles into microbial therapies for wildlife. Spheres represent microbial communities (microbiomes), and their trajectories reflect changing community structure and/or function.

Given the complexity of interactions between hosts and their associated microbiota, there are numerous ecological processes to consider when formulating microbial therapies. We briefly summarize three sets of ecological processes that are particularly relevant to understanding how microbial therapies may elicit beneficial phenotypes or outcomes: (i) colonization and succession ecology, (ii) resistance, resilience, and stable states, and (iii) functional redundancy and supplementation. These three are not mutually exclusive and are, in part, interdependent. For example, we discuss functional redundancy as a distinct concept but also as a component of colonization and ecological stability. These three concepts also encompass numerous other ecological processes, which we refer to throughout the text. We suggest that these three sets of ecological principles provide a framework for understanding how to manipulate animal microbiomes (Fig. 1).

### Colonization and succession ecology

Colonization in animal microbiomes can occur for many reasons, both adaptive and maladaptive. These processes include early-life microbial assembly, pathogen infection, and purposeful colonization by therapeutic microbes. Regardless, the succession of colonization and future community assembly is shaped by niche partitioning, availability of resources, and competitive exclusion. If a stable microbial community has high resource competition and utilization, the likelihood of colonization is low given minimal open niches (21, 22). In contrast, if a community has high resources and low competition, it may be susceptible to novel colonization and subsequent niche partitioning by colonizing microbes (21, 22).

Early-life microbial colonization in animals occurs in the context of open niches, the absence of strong competition, and species-specific physiological and immune development, facilitating the relatively rapid development of host-specific microbial communities (23). For example, in vertebrates, maternal seeding of neonatal communities occurs rapidly through birth and subsequent exposures (24), such as through nursing (25), parental care (26, 27), and grooming (28). This early and rapid colonization has been shown to facilitate early-life immune imprinting and provides a key microbial community that resists pathogens in immune-naïve neonates (29). Priority effects are also notable in their effects on subsequent host microbiomes and probiotic colonization, in which timing of exposure can have different outcomes depending on which microbes have already colonized (30, 31).

Colonization success is further shaped by the ecology of the colonizer paired with environmental, physiological, and genetic conditions of the host (32). Pathogenic infection can occur in the context of opportunistic niche exploitation as well as competitive advantages (e.g., antibiotics, toxin production, or immune system evasion). Opportunistic pathogens are resident members of microbial communities that expand their colonization into a detrimental infection, usually in response to a lessening of competitive exclusion or a novel boost of relevant resources. For example, a mass mortality event in saiga antelope (*Saiga tatarica*) was attributed to the prevalent commensal *Pasteurella multocida* serotype B, which proliferated under ideal environmental conditions (high heat and humidity), leading to infections of the upper respiratory and gastrointestinal tracts (33). In humans, *Staphylococcus epidermidis* is a known skin commensal that plays a role in maintaining skin defenses and immune interactions (34). However, *S. epidermidis* is also a common cause of nosocomial infections due to its propensity to form inflammatory biofilms and evade antibiotic treatment (34), highlighting the importance of condition-dependent colonization potential. Host physiology, such as immune system properties, and host genetics can further drive colonization ecology (35, 36). In humans, the effect of both high-level genetic patterns and single genetic variants on microbial communities varied across body sites, indicating that host genetic make-up exerts distinct colonization and maintenance pressures across specific communities (37). By incorporating studies of colonization and succession ecology into models of microbial therapies, we can better predict which microbes and environments promote consistent host-microbe symbiosis.

### Ecological stability and resilience

Resiliency dictates whether a community can adapt after a perturbation and return to a similar stable state. The microbiomes of healthy hosts are generally considered resilient, with communities returning to stable states with similar, but perhaps not identical, structure and function following changes such as seasonal or environmental variation (38). Occasionally, however, host-associated microbial communities undergo perturbations that shift the community into alternative stable states that are still adaptive to the host. The effect of weaning in mammals (39, 40), temperature shifts linked to pathogen risk in amphibians (41), heat tolerance in reptiles (42), and migration in birds (43) are examples of complex perturbations that usually resolve into an alternative community with novel structure and/or function that are adaptive to the host’s current condition. The resilience of a host-associated microbial community will also determine the need for therapeutic interventions. Importantly, persistent perturbation can increasingly diminish community resilience, resulting in increased need for microbial therapies to assist in the recovery of a stable state (44, 45).

For animal microbiomes, there are myriad opportunities for perturbations that are either natural (e.g., seasonality, life-history transitions, disease) or anthropogenic (e.g., antibiotics, pollution). These perturbations can be focused—such as precision antibiotics eliminating a single pathogen (46, 47)—or broader in scope—such as the effects of seasonality or diet changes (48–50). It is well established that the microbiota of wildlife under human care differs from those of wild conspecifics (13, 51–53). Although the reasons for these differences are multifaceted, some research indicates lower community resilience in *ex situ* wildlife, as evidenced by increased perturbation potential (e.g., from antibiotic use), higher incidence of chronic disorders, and greater vulnerability to pathogens (13, 51). Moreover, community stability and resilience vary across *ex situ* species, with some host species showing high inter-individual variation responses to the same perturbation and other species showing conserved responses across individuals (48). Understanding the stability and resilience of human microbiomes is recognized as a key component of microbial interventions for human health (54). The same is true for animals; studying how animal microbiomes maintain stability and resilience under varying perturbations will inform successful microbial therapies.

### Functional redundancy and functional supplementation

A core characteristic of many complex microbial communities is functional redundancy—the maintenance of multiple taxa with the same functional capacity. This is exemplified in vertebrate gut microbiomes, where functional redundancy is hypothesized to be critical for the maintenance of host-microbe symbiosis (55). Functional supplementation occurs when a taxon can provide a function that is novel and advantageous to the community, enabling it to be incorporated into the community. Functional supplementation may also provide capacity that was previously lost due to perturbation and can therefore be ephemeral, acting as a potential stopgap during periods of perturbation.

Both functional redundancy and supplementation are facets of the two ecological processes discussed above. Functional redundancy can underlie ecological resilience such that if some microbial taxa are eliminated, the community does not suffer a loss of function and remains adaptive. In a cross-biome meta-analysis of microbial communities, including cattle gut microbiomes, researchers observed a high prevalence of functional redundancy, pointing to a relationship between functional redundancy and environmental adaptation (56). In the skin microbiome of red-backed salamanders, the distribution of bacteria shown to kill the fungal pathogen *Batrachochytrium dendrobatidis* was found to be functionally redundant across varying habitats, despite varying taxonomic compositions and environmental conditions (57). Yet, the concept of functional redundancy is not without nuance. In a metabolomic examination of the same pathogen-microbiome interaction but a different amphibian host, the authors found no support for functional redundancy as the metabolome correlated strongly with bacterial taxonomy (58). Therefore, in some instances, functional supplementation could be a useful microbial therapy conservation strategy.

Functional supplementation may also promote colonization through the selective seeding of novel functional groups. It is hypothesized that vertebrate species acquire symbionts through environmental acquisition, filtering out diverse environmental microbes and allowing colonization by taxa that likely provide functional benefits (59, 60). If environmental conditions change, animals may be able to functionally supplement their microbiome. For instance, in vultures, the acquisition of novel microbes from carcasses is hypothesized to provide critical microbial defenses and functions in skin and gut microbiomes (61, 62). However, the process of functional supplementation in complex microbial communities such as vertebrate microbiomes is poorly understood, and much needs to be learned before this concept can effectively inform conservation goals (63).

The functional capacity of wildlife microbiomes is an area of much interest, but there are still significant knowledge gaps. Maintaining functional redundancy and applying functional supplementation are target goals of many microbial interventions. Yet, interpretation of microbial function varies according to the methodology (e.g., metagenomics, metabolomics, meta-transcriptomics) and the target outcomes (e.g., host health, reproduction, dysbiosis). Moreover, the degree of functional redundancy or supplementation needed to accomplish a specific outcome and the associated microbial taxa are not known for most animal species. Even basic functional profiling is lacking for many wildlife species, which presents a challenge to the application of microbial therapies. The study of these ecological principles in wildlife, particularly in relation to microbial function, will provide critical context for manipulating animal microbiomes and health outcomes.

## APPLYING MICROBIAL ECOLOGY TO MICROBIAL THERAPIES IN WILDLIFE

The concepts discussed above are particularly relevant to three types of microbial therapies that are widely used in wildlife care and conservation: prebiotics, probiotics, and whole-community transplants. These therapies are usually applied in response to perturbation, when a microbial community is in a state of dysbiosis, dysfunction, or change due to an environmental pressure (e.g., pathogen invasion). Although less common, microbial therapies can also be used as prophylactics to prevent dysbiosis, dysfunction, or change. Below, we define these therapies and synthesize how microbial ecology can inform and advance each treatment type in wildlife. Within an ecological framework, we discuss how these microbial therapies could act either as a prophylactic to resist negative change or, more commonly, as a therapeutic in response to perturbation.

### Prebiotics

Prebiotics are dietary or nutritional components that promote the maintenance and propagation of beneficial microbes. Prebiotics often require microbial fermentation or processing to produce bioavailable nutrients or compounds that would otherwise be unavailable to the host (64). Prebiotics in wildlife species can span the gamut of nutritional options, reflecting the diversity of dietary ecologies. In wildlife under human care, there is increasing adoption of prebiotic dietary components as tools to “rewild” animal diets and lessen related health concerns, a concept grounded in evolutionary medicine (13). For example, frugivorous primates under human care were traditionally fed fruits cultivated for human consumption, which are significantly higher in sugar and lower in fiber than natural fruits consumed by wild conspecifics. This discrepancy resulted in a high incidence of diet-related health issues in *ex situ* primates such as diabetes, obesity, and dental problems (13). Additional examples across wildlife species include providing browse that diversifies complex plant fiber for herbivorous species (65) and whole carcasses that provide animal fibers (e.g., fur, tendons, cartilage, bones) for carnivores (66, 67). Given the importance of resource availability in shaping microbial communities, prebiotics can facilitate colonization, increase community resilience, and provide functional supplementation.

Prebiotics that promote the colonization, succession, and niche partitioning of host-specific beneficial microbes and functions can improve microbial symbiosis and facilitate recovery following microbial perturbations. A recent study in mice demonstrated that appropriate dietary resources (i.e., low-fat, high-fiber chow) facilitated the post-antibiotic colonization of gut microbiota composition and function, outperforming FMT treatment in recovery trajectories (68). Similarly, prebiotics can facilitate the maintenance and transition to stable community states. In a study of prebiotic diets in five frugivorous monkey species housed in similar environments, researchers replaced fruits with nutritionally balanced vegetables to reduce sugar intake and increase prebiotic fiber intake (49). The diet shift resulted in an increase in abundance of bacteria that break down complex plant fiber (e.g., *Phascolarctobacterium*, *Megasphaera*, and *Sharpea*) (49). Interestingly, despite nearly identical management conditions and identical prebiotic dietary manipulations, the fiber-degrading taxa that responded to the increased dietary fiber were host-specific across the five species, suggesting host-specific alternative stable states. Prebiotics can further facilitate functional supplementation through increased resource availability for microbes that were reduced or extirpated due to perturbation (i.e., disease). In an Asian elephant suffering from colic, supplementation with prebiotic inulin—a plant polysaccharide—shifted gut microbiome composition, improved gastrointestinal health, and alleviated symptoms (69). Health outcomes of the elephant inulin treatment were confirmed via FMT in gnotobiotic mice, which showed that the ‘inulin microbiota’ promoted intestinal cell proliferation, increased short-chain fatty acids utilization, and reduced inflammation (69).

For prebiotics in particular, consideration of host ecology and evolution is particularly important for practical applications—dietary or nutritional “mismatches” can drive microbial dysbiosis and negative health outcomes (13). Ecological principles can thus be practically integrated into prebiotic formulation by (i) identifying resource (nutrient) requirements that maintain the resilience of wildlife microbiomes, (ii) determining how niche partitioning across microbes drives resources availability within wildlife microbial communities, and (iii) applying appropriate resources in the form of nutrients or diet items that best recapitulate the evolved diet of the given wildlife species.

### Probiotics

Here, the term “probiotics” refers to supplemental live microbes that are purported to have beneficial health impacts. Different probiotics can have distinct mechanisms of action, including immune modulation (70, 71), the production of beneficial metabolic byproducts (72), or direct microbial interactions such as competitive exclusion (73, 74). It is, however, important to distinguish the use of probiotics in medical treatment (e.g., prescription drugs) versus their use in commercial industries (e.g., dietary supplements). Commercial probiotics for humans are now widely available as standalone supplements, food additives, and components of beauty and lifestyle products. For medical probiotics, however, there is increasing research supporting their use in disease treatment (75). These studies, however, have also highlighted significant variability in treatment efficacy across disease and individuals (75–77). For example, probiotic efficacy can vary significantly by factors such as probiotic strain specificity (77) and an individual’s native microbiome (78, 79). This has led medical researchers to call for a “personalized medicine” approach to medical probiotics in humans (80). Despite the more tailored approach being adopted in human medicine, most probiotics for animal care can still be categorized as commercial supplements with little empirical support (81). Commercial animal probiotics are rarely formulated according to the host’s microbial ecology, with most probiotic taxa still being of human origin despite being marketed for use in animals (81). Exceptions to this include probiotics being developed for the mitigation of specific wildlife infectious diseases, such as chytrid disease in amphibians and white-nose syndrome in bats (7, 9). These examples mirror the more “personalized” approach to probiotics that is emerging in human medicine, with probiotic strains being isolated for host-specific or pathogen-specific beneficial functions. Nevertheless, supplemental commercial probiotics are widely used in wildlife care and conservation, with some limited evidence for health benefits (9, 82).

A common hallmark of probiotic efficacy is the degree and persistence of colonization by the probiotic strains (83). Among humans, the colonization of a probiotic strain can vary widely across individuals and is, in part, dictated by the resident microbiota (78, 79). However, probiotic colonization has also been shown to compete with and even hinder the recovery of native microbiota following perturbation (e.g., post-antibiotic recovery), indicating that, in some cases, probiotic colonization can decrease community resilience (84, 85). This is relevant to commercial probiotics used in wildlife because the probiotic strains (i) are unlikely to be native members of the microbiome, (ii) have not been empirically tested in the target host species (wildlife), and (iii) may be taxa that have been shown to successfully colonize human microbiota, which is not a characteristic that would necessarily benefit wildlife microbiota.

Certain mechanisms of probiotic action do not necessarily rely on sustained colonization. Probiotics have been identified and tested for wildlife disease management using the concepts of ecological stability, resilience, and functional supplementation. Notably, infection by wildlife pathogens leads, in most cases, to an altered microbiome at the infection site, indicating localized shifts in community stability. These include chytridiomycosis in amphibians (58, 63), white-nose syndrome in bats (86, 87), snake fungal disease (88), and multiple pathogens in invertebrates such as corals (89) and bees (90). Researchers in these systems generally isolate bacteria from the body region affected by the pathogen, identify the bacterial strains that can kill the pathogen *in vitro*, and then make decisions about which bacterial strains to apply to the host in infection experiments. We suggest that determining which pathogen-inhibitory bacterial strains are found to be important in microbiome community stability and that interact in non-detrimental ways with the host immune system (1, 58, 70, 91) will lead to improved effectiveness of probiotics. Synergistic effects of pathogen inhibition can occur among bacterial strains, suggesting that mixed-strain probiotics have the potential to be more effective than single-strain probiotics (92, 93), given the increased probability of beneficial ecological interactions.

We suggest that the integration of microbial ecology into the practical application of probiotics can be achieved through two key steps. First, identify the need for probiotics—for example, prophylactic to maintain homeostasis vs. therapeutic to support recolonization after perturbation or to combat disease. Second, identify and apply relevant, host-specific probiotic candidates that can fulfill the target need. This second step may be particularly difficult given the relative lack of literature on the function of wildlife microbiomes—yet we emphasize that this is a key component of wildlife microbial ecology that underpins the value of probiotics. Combined, these determinations will shape practical decisions such as dosage, frequency, and monitoring.

### Whole community transplants

Whole community transplants consist of transferring a specific microbial community to either the same individual (i.e., autologous transplant) or to a conspecific individual (i.e., allogenic transplant). Fecal microbiota transplants (FMTs) are the most common method of whole community transplant and are used, primarily in mammals, to address gastrointestinal issues (94). In its simplest form, an FMT is comprised of whole feces that have been screened, deemed safe, and encapsulated or processed for transplantation. Although FMTs are not without risk and are often considered a last-resort treatment, they have shown success in treating otherwise intractable diseases and infections in humans and animals (94–96). Most widely known for their use in treating *Clostridioides difficile*, FMTs are additionally being used in humans to treat other GI concerns (irritable bowel disease), certain cancers, and psychological disorders (97–99).

Other community transplants have additionally shown promise as treatments for health concerns in humans and animals. For example, vaginal microbiome transplants are newly recognized as a treatment for bacterial vaginosis in humans (100, 101). Similarly, in humans, oral microbiota transplants can alleviate periodontal disease (102, 103). In animals, rumen microbiome transplants have a long history in the care of ruminants (104, 105), and skin microbiome transplants are being explored as a novel treatment for fungal infection in amphibians (91).

Currently, whole community transplants are the microbial therapies that are most well-grounded in ecological frameworks, reflecting the need to treat the entire host-microbe ecosystem rather than individual components. Transplanting entire communities of microorganisms and associated molecules may best minimize instability and recreate a stable state. In other words, an important mechanism of microbial therapies may stem from the collective action of entire communities of microbes, not just from one or two strains (13).

There is growing empirical support for FMT use in wildlife across varying life history stages, conditions, and ecologies. Whole community transplants have been used in humans and other mammals to promote early-life colonization (106, 107). Specifically, FMT from adult fecal matter into mammalian neonates has been used to promote initial microbial colonization in cases where neonates are diseased or lacking natural conspecific microbial exposures (108), although most uses in wildlife are anecdotal (13, 109, 110). FMTs have further been shown to improve community resilience. In lemurs and cheetahs, FMT following antibiotic treatment reduced community instability and hastened the recovery of microbial consortia that resembled pre-antibiotic communities (45, 111). In a two-toed sloth (*Choloepus didactylus*) experiencing abnormally frequent and loose stools, administering multiple FMTs of donor feces from a co-housed conspecific alleviated the abnormal defecation and altered the recipient’s microbiome, likely shifting it into an alternative stable state (112). FMTs have additionally been shown to promote functional supplementation. In desert woodrats, FMTs from donors that successfully digest toxic plant compounds provided the recipient animal with microbes that increased digestive capacity and detoxification (113). Likewise, in koalas, FMTs from donors eating a particular type of *Eucalyptus* lead to the recipient animals being able to consume that as a new dietary item (114).

We suggest that the application of FMTs can be informed by ecological principles through the (i) incorporation of colonization ecology and turnover rate into FMT dosing and administration, (ii) use of donor material that represents resilient, stable state to provide the best likelihood of engraftment and stabilization, and (iii) identification of FMT communities that may provide functional supplementation, either as a stopgap or as a component of recovery to an alternative stable state.

## FUTURE DIRECTIONS FOR MICROBIAL THERAPIES IN NON-MODEL ANIMALS

Fostering animal health under human care, in reintroductions of endangered species, and in wild populations is critical to combating ongoing losses of biodiversity. Although microbial therapies can offer a useful conservation strategy to improve reproductive health, promote a healthy gut-brain axis, and combat disease, they require rigorous empirical research to be successful. There is a growing need for multidisciplinary experts to interpret and integrate microbial ecology and microbiome research into practical animal health and welfare. Given the sheer diversity of microbial taxa living in and on animals, we must ask, “What do we focus on and how do we do it?” We conclude this minireview with practical considerations to address these questions and offer insight into what the future of conservation microbial ecology will look like as the field rapidly progresses. We first discuss the need for robust, longitudinal sampling across wildlife populations both under human care and in the wild, with a focus on functional profiling. Our next directions on prebiotic usage and whole community transplants are targeted more specifically for animals that are under human care or that will be reintroduced into the wild. Our final directions are focused on developing effective probiotics that can be applied more broadly and on harnessing novel technologies such as artificial intelligence (AI) to identify patterns across complex microbial data sets.

### Advancing study designs to characterize wildlife microbial ecology

Although wildlife microbiome research is burgeoning (6, 9, 10, 13, 115–117), the field has some critical knowledge gaps that have limited its applied value. These gaps are largely due to the challenges inherent in studying wildlife and the costs associated with larger, more complex studies. Nevertheless, the first series of future directions we suggest are components of study design that can advance the field as a whole.

- Incorporating longitudinal monitoring of wildlife microbiomes can elucidate patterns of stability, plasticity, and turnover, providing key insights into how microbial communities change over time, with host and environmental variables, and in response to microbial therapies.
- Using methods that characterize microbial function alongside taxonomic identity (e.g., multi-omic approaches) will greatly expand our understanding of host-microbe symbiosis across diverse wildlife species.
- Performing non-invasive experimental manipulations—or using “natural experiments”—is necessary to characterize causative effects and mechanisms, which are particularly important for microbial therapy interventions.
- Increasing representation of wild animal populations in microbiome studies—while continuing to study populations under human care—can better contextualize the diversity of microbiomes that can support and maintain host health.
- Pairing microbiome studies with host and/or environmental health metrics will promote interdisciplinary and “One Health” perspectives on microbial ecology and will bridge the current divide between basic microbial ecology and practical wildlife care and conservation.

### Practical and accessible microbial therapies for wildlife under human care

For wildlife under human care, there are three future directions that have particular applied value. First, prebiotics in the form of diet changes can be some of the quickest strategies to implement in the treatment of gut-related issues. Diet has been shown to impact gut microbiomes in many *ex situ* species (14, 49, 66, 118–122). However, given that a low sample size is inherently a challenge in endangered species research, it has been challenging to link diet-related changes in animal microbiomes to impacts on the health of the individuals. Wildlife care facilities (e.g., zoos) often have only a few individuals of a given species, and each facility provides different diets and environments, making it difficult to tease apart the specific influence of diet versus environment on animal microbiomes and health. Furthermore, gut microbiomes can be inherited via colonization of maternal or parental microbes (123), potentially resulting in more pronounced generational effects in the gut microbial health of wildlife under human care. We emphasize that diets of wildlife under human care should be formulated to consider prebiotics and the microbial ecology of animal gut microbiomes (13). Given the environmental drivers of microbial colonization, resilience, and function that we discussed above, we suggest that greater discussion in conservation venues (e.g., Species Survival Plan or AZA SAFE meetings) would encourage facilities to combine forces toward understanding environmental and dietary influences on animal microbiomes and health.

Second, whole microbiota transplants offer a rapid and cost-effective strategy to explore, with primary application for animals under human care. Examples of whole community transplants that could be applied to animals in human care, but currently remain solely in the human medicine arena include (i) vaginal microbiota transplants to inoculate babies born via cesarean section and for treatment of bacterial vaginosis (100, 101), (ii) skin microbiota transplants for dermatological conditions such as eczema (124), and (iii) oral microbiota transplants for dental decay and periodontitis (103). For these treatments, however, the microbial agents involved in this process are largely unknown. From a practical standpoint, however, it may not be critical to know the exact mechanisms of action for the treatment to be effective. Yet, concerns exist regarding their safety (e.g., undetected pathogen or antimicrobial resistance genes) and standardization (e.g., ensuring consistent beneficial effects across different donor materials). Nonetheless, in human FMT research, these risks have been determined to be minimal in comparison to the widespread benefit in the treatment. For example, two microbial-based products derived from human fecal donations (Rebyota and Vowst) have been approved by the US Federal Drug Administration to treat *C. difficile*. These products contain complex microbial communities for which the whole community is the treatment, and no individual microbial members are disclosed or approved individually. Recently, Tian et al. identified the functionally relevant taxa (proline-fermenting strains such as *Peptostreptococcus anaerobius*) that underlie the success of FMTs in treating *C. difficile* infection in humans (1). We see the potential for future work in conservation microbiomes to identify the underlying functionally relevant taxa for which probiotics can then be developed. But this process requires time and money, which can be limited in conservation. For recurrent GI issues in animals under human care, FMTs from a healthy conspecific are currently one of the best treatment options. There is an effort to promote the biobanking of healthy fecal material for use in wildlife FMTs, which has already proven useful for the conservation and welfare of certain species (111). However, given the wide range of microbial physiologies and requirements present in communities used for transplantation, long-term preservation of these complex microbial communities poses a challenge for human and animal medicine. Moreover, the effective dosage and frequency of whole community transplants that would be needed to treat conditions across wildlife species is largely unknown. We reiterate the need for microbial ecology concepts to be integrated into future investigations of whole preservation, dosage, and monitoring of whole community transplants.

Third, action should focus on microbial cultures and the development of probiotics if whole community transplants are not feasible. The first step here is isolating bacteria—or other microbes such as fungi—from the animal species and body site of interest. We acknowledge that fungi can play an important role in animal ecology and health (125, 126), but the resources for fungi are more limited (e.g., limited databases and knowledge of culture growth needs), leading to a currently limited scope of use. Cross-domain interactions between bacteria and fungi can have important effects on health (127), and we anticipate greater attention to these interactions as high-throughput sequencing methods improve, leading to the identification of important fungi for which targeted culturing can be used (128). Currently, bacteria are the leading agents in human and wildlife probiotics (129). Yet, commercial probiotics have generally been developed with limited consideration of host ecology and evolution. As we argue here, the future for successful application of probiotics requires consideration of host biology and the development of probiotics that can be used in multiple host species effectively. Perhaps one of the best examples of probiotic development for conservation efforts is the application of bacteria known to kill the fungal pathogen *B. dendrobatidis* onto amphibian skin (63, 82, 130) or into the amphibian’s environment (131), as well as similar parallel efforts to treat bats infected with the fungal pathogen *Pseudogymnoascus destructans* (128). In these efforts, probiotic success is exceedingly difficult to predict, and each probiotic formulation interacts differently with the host (70). If the target outcome of the probiotic is not to kill a pathogen, but to mitigate some other host health concern (e.g., treating reproductive dysfunction), then greater data on the mechanisms of action are needed. Meta-analyses have been key in human research advancements. To accelerate the pace of identifying the best microbial strains that will colonize the host and serve the desired function, we see the future needing (i) more meta-analyses or large-scale comparative studies to identify broad-spectrum bacterial symbionts (either occurring in multiple species affected by the same condition and/or occurring across multiple facilities/sites with the same species), (ii) development of organ-on-a-chip models (132–134) for wildlife to reduce the need for animal trials, and (iii) an engineering microbiome approach as described next.

### Engineering and studying microbiomes in a big-data world

The engineering of microbiomes is becoming a widely researched concept in human medicine (135–137) and agriculture (138–140) and can be applied to wildlife conservation questions. These approaches focus on building synthetic microbial communities (SynComs) using either mixtures of single-strain cultures (bottom-up) or multi-strain mixtures isolated from complex microbiomes (top-down) to identify functionally relevant multi-strain communities. In human medicine and agriculture, these studies are often paired with whole-community DNA sequencing data to determine the distribution of the members of the SynComs in the native host microbiome (1, 141). For a top-down approach, one could take samples from animals resistant or susceptible to a pathogen, grow the microbial communities from those samples in a medium to create a synthetic, culturable representation of the whole community, and then add the pathogen to those communities to determine their effects on pathogen colonization. If the resistant samples show a reduction in pathogen colonization, then future work can isolate the individual strains involved in that function. For a bottom-up approach, strains known to serve a desired function and/or shown to be central nodes in whole-community ecological networks and correlated to disease outcome could be added together to determine if they have effects on pathogen colonization, such as experiments done with the amphibian pathogen *B. dendrobatidis* (142) and with human pathogens *Klebsiella pneumoniae* and *Salmonella enterica* (135). For non-pathogen-associated issues (e.g., inflammatory bowel disorder, reproductive dysfunction), bottom-up and top-down approaches have been applicable for study in microplates (143, 144), in bioreactors (145), and ultimately in mouse models (146) before advancing to human medicine applications. Mouse models have been used in non-model animal microbiome research (147, 148) and may offer a system to test hypotheses about new microbial therapeutics before use in endangered species, although we caution that the species-specific nature of microbiomes may hinder progress if not considered (149).

Finally, the rapid and extensive generation of “multi-omic” data warrants the use of “big-data” tools to identify patterns of microbial dynamics that could signal successful microbial therapies. AI, machine learning, and deep learning methods can detect patterns in complex and non-linear data, but such tools also require high-quality training data sets (150). Importantly, these analytic tools are also capable of integrating non-microbial data sets, allowing for the synthesis of microbial and host data to predict outcomes (151). Neural networks, for example, have been used to identify microbe-metabolite interactions in both environmental (soil) and human clinical data sets (152). Pitfalls of these tools include challenges in reconciling varying methodological approaches (e.g., sample collection, metadata, and sequencing protocols) (153). This is particularly relevant to wildlife microbiome research, for which standardized protocols have yet to be implemented, and metadata reporting varies widely across studies. To enable the application of analytic tools, we suggest that wildlife microbiome researchers consider adopting the STREAMS protocols (Standards for Technical Reporting in Environmental and Host-Associated Microbiome Studies) (154), which address challenges specific to environmental and non-human host-associated studies.

In regard to microbial therapies for wildlife, future data-oriented approaches, combined with the study designs discussed above, have specific relevance to detecting or predicting the following:

- conserved or host-specific microbial patterns across comparative data sets from numerous host species,
- patterns of stability and colonization across animal life histories using longitudinal data sets,
- disease-specific processes and dynamics across vulnerable or resistant host species,
- microbial responses to changing environmental conditions (e.g., climate change), and
- microbial mechanisms of host health via the integration of veterinary and nutrition data sets.

Together, these approaches can guide priorities for developing either precision microbial therapies (i.e., to treat disease-specific processes) or more generally applicable therapies that can be used across species (e.g., to maintain general health or combat common ailments).

## CONCLUSION

Overall, microbial therapies are gaining attention for their application across human, animal, and industrial sectors. Human microbiome research is embracing the value of microbial ecology in developing microbial therapeutics, a practice that could greatly benefit the study and manipulation of wildlife microbiomes. The formulation of microbial therapies to treat specific wildlife infectious diseases has highlighted the need for increased integration of interdisciplinary research on host biology and microbial ecology. Understanding the colonization, stability, resilience, and function of wildlife microbiomes will underpin the development of successful microbial therapies. Moreover, innovations currently being developed for human medicine (e.g., precision probiotics, engineered microbiomes, and AI detection of microbial dynamics) have significant potential to improve the care and conservation of endangered species. However, microbial therapies do have pitfalls and are not “panaceas” for human or animal health concerns—manipulating microbiomes may only be beneficial if there is a substantial microbial component to the underlying issue. Especially now, when there is minimal standardization and we are only starting to generate empirical evidence in wildlife species, microbial therapies should be carefully considered to ensure safe and appropriate use. Additionally, there is a need for balance between feasibility and practicality that will dictate the development and application of microbial therapies for wildlife. Future research should not only incorporate microbial ecology principles but also consider the feasibility (e.g., accessibility of treatment components, use as prophylactic or therapeutic) and practical applications (e.g., preservation, dosage, frequency, use across many species) of microbial therapies. Future work needs to promote microbial therapy development for wildlife conservation by (i) including longitudinal, non-invasive, yet experimental studies, (ii) integrating big-data or AI approaches to identify conserved or unique microbial patterns across species, and (iii) emphasizing financial investment in the formulation of accessible microbial therapies.
